# Development of Pty-co-SAXS: Combined Ptychography and Small-Angle X-ray Scattering at APS 12-ID-E

**DOI:** 10.1063/4.0000880

**Published:** 2025-10-27

**Authors:** Byeongdu Lee, Soenke Seifert, Joseph McCourt, Junjing Deng

**Affiliations:** Argonne National Laboratory, Lemont, IL, USA

## Abstract

X-ray diffraction techniques, including both small- and wide-angle scattering, are valuable for scanning imaging when samples contain molecularly packed structures. These structures offer dark-field imaging contrasts, which are useful for visualizing various biological fibers such as collagens, axons, and muscles. Utilizing these contrasts, tomograms exceeding three dimensions can be reconstructed, exemplified by SAXS tensor tomography. Typically, dark-field imaging requires complementary bright-field imaging for a comprehensive understanding of the materials.

Recently, we have developed a beamline, 12-ID-E, for simultaneous SAXS and ptychographic imaging. This instrument not only provides both dark and bright field imaging simultaneously but also captures multi-scale pictures of hierarchical structures. The beamline is equipped with two Pilatus detectors and a hexapod-based ptychography setup. In this presentation, we will detail the setup and showcase scientific examples involving the self-assembly of nanoobjects.

